# Comparison of Knowledge and Attitudes of Medical and Dental Students towards HIV/AIDS in Pakistan

**DOI:** 10.7759/cureus.2426

**Published:** 2018-04-04

**Authors:** Asad Ali, Nouman Safdar Ali, Usama Nasir, Muhammad Aadil, Neha Waqas, Ahsan Zil-E-Ali, Muhammad Jahanzaib Anwar, Ibrar Anjum

**Affiliations:** 1 Medicine, CMH Lahore Medical College and Institute of Dentistry, Lahore, PAK; 2 Department of Medicine, Jinnah Hospital, Allama Iqbal Medical College, Lahore, Pakistan; 3 Department of Medicine, FMH College of Medicine and Dentistry; 4 Surgery, Shaikh Khalifa Bin Zayed Al Nahyan Medical & Dental College, Shaikh Zayed Medical Complex, Lahore, Pakistan. 54600, Lahore, PAK; 5 General Surgery, Fatima Memorial Hospital, Lahore, PAK; 6 Department of Internal Medicine, Rush University Medical Center; 7 Internal Medicine, The University of Texas MD Anderson Cancer Center, Houston, USA

**Keywords:** health care professionals, medical students, dental students, hiv/aids, attitude, awareness, knowledge

## Abstract

The number of new human immunodeficiency virus (HIV) cases is increasing in Pakistan while it has seen a decline globally. A survey conducted recently has found that 132,000 people in Pakistan suffer from HIV. This study aims to check the levels of knowledge and attitudes about HIV/acquired immunodeficiency syndrome (AIDS) among medical and dental students.

This cross-sectional study was designed and conducted at Combined Military Hospital Lahore Medical College and the Institute of Dentistry (CMH LMC) in Lahore, Pakistan in 2016. Students enrolled in the courses of the MBBS and BDS were included in this study. The questionnaire consisted of three sections: demographics, knowledge and attitude.

A total of 414 students completed the questionnaire and out of them, 286 were medical students while the rest were dental students. The mean ± standard deviation score for the students was 10.02 ± 4.37 out of 17 for knowledge related to HIV and AIDS. For acceptable attitude towards AIDS and patients afflicted with the disease, an outcome of 1.93 ± 0.75 out of 4 was observed.

The results of this study indicate lack of knowledge about HIV, especially about the modes of transmission and prevention techniques. Therefore, regular interactive workshops and seminars, besides teaching sessions, focused lectures on HIV/AIDS, need to be conducted.

## Introduction

Human immunodeficiency virus (HIV) that causes acquired immunodeficiency syndrome (AIDS) is a social disease that has become a global pandemic within three decades since its discovery, affecting more than 36 million people worldwide. Of the estimated, 36.7 million people infected with HIV in 2015 and 1.8 million are less than fifteen years old [1]. Although the prevalence in children is low, it is on the rise [2]. Underdeveloped countries carry the burden of 95% of total infection and 90% of total deaths due to HIV/AIDS. Despite our better understanding of the disease, there is still no cure for the disease. Though antiretroviral therapy (ART) exists, it does not eradicate the virus. It merely inhibits the virus from replicating within the body hence halting the progression of the disease and increasing the number of people living with HIV/AIDS (PLWHA). Therefore, more healthcare professionals are encountering PLWHA in their careers.

Pakistan Economic and Labour Survey 2014-15 estimated the population of Pakistan at 191,715,847. Of this population, 45.3% is illiterate, and more than half of these people live in villages where educational opportunities are deficient. United Nations AIDS (UNAIDS) - National AIDS Control Program of Pakistan 2014 estimated the prevalence of HIV/AIDS to be less than 0.1%. The population has poor health-seeking behavior [3]. Integrated Biological and Behavioral Surveillance (IBBS) 2016-17 in Pakistan identified the high-risk populations that need to be addressed to combat the growing epidemic of HIV infection. IBBS described concentrated epidemics in these populations and reported weighted prevalence estimated as follows:

1) Patients who inject drugs (PWID) 38.4% (95% CI: 37.9–38.9)

2) Transgender (TG) 7.1% (95% CI: 6.8–7.4)

3) Female sex workers (FSW) 2.2% (95% CI: 2.1–2.3)

4) Men who have sex with men (MSM) 5.4% (95% CI: 5.2–5.6)

When compared to previous reports, FSWs showed the highest rate of increase in HIV infection. Lack of knowledge and poor prevention attitudes including the use of condoms were recognised as major risks for increasing burden of the disease. A recent systematic review also identified migrants, prisoners and multi-transfused patients with high prevalence of HIV [4].

UNAIDS puts forward a holistic combination prevention approach for HIV prevention. Combination prevention as stated by UNAIDS is “rights-based, evidence-informed, and community-owned programs that use a mix of biomedical, behavioural, and structural interventions, prioritised to meet the current HIV prevention needs of particular individuals and communities, to have the greatest sustained impact on reducing new infections.” Behavioural changes and appropriate education provide a strong foundation for this prevention strategy. Increasing awareness related to HIV can be vital to achieving 2030 HIV elimination goals set by UNAIDS for Pakistan [5].

It is the responsibility of healthcare professionals to provide these patients with updated education and counselling so that they remain appropriately functional in their lives. Physicians and dentists make up a major chunk of healthcare professionals. Sufficient knowledge and positive attitude of medical as well as dental students towards HIV/AIDS are of utmost importance. Medical and dental students equipped with sound knowledge of the disease and resultant professional attitude will be better able to take good care of the patients and themselves. A better understanding of the students’ prior knowledge and attitudes towards HIV/AIDS can serve as a tool to create better educational programs, combating stigmas and taboos, and encouraging empathy towards the patients dealing with HIV and AIDS.

## Materials and methods

A cross-sectional survey was conducted at Combined Military Hospital Lahore Medical College and the Institute of Dentistry (CMH LMC) in Lahore, Pakistan in 2016. Study approval obtained from the CMH Lahore Medical College Ethics Review Committee. Data was collected using a well-structured questionnaire which distributed among potential respondents (medical and dental students at the college). A sample size of 414 was obtained which included medical students (286) and dental students (128). Informed consent was obtained from every individual included in this study.

Data collection form with both open ended and close ended questions used. The survey consisted of three parts. The first part had demographic details, including the designation, age and gender. In the second part, 17 questions were asked to determine the level of knowledge about HIV/AIDS among students. Third part consisted of four questions regarding the attitude of students towards the disease. The correct response scored as one and an incorrect or no responses as zero. Participants were told the aim of this survey and assured of the strict confidentiality of their responses. Informed consent was obtained from questionnaire administration.

We analyzed the data gathered from a preformed questionnaire using SPSS version 21.0 (IBM SPSS Inc., Chicago, IL, USA). Based on the outputs, we calculated the means and standard deviations for knowledge and attitude scores. Data was analyzed for normalcy using skew and kurtosis cut-offs while homogeneity was assured via Levene’s F test. Variables were analyzed using frequencies, percentages, and independent samples t-tests. A p-value less than 0.05 was considered significant.

## Results

Of the 414 students who completed the questionnaire, 286 were medical students while the rest were completing their dentistry program. The mean ± standard deviation score for the students was 10.02 ± 4.37 out of 17 for knowledge related to HIV and AIDS. For acceptable attitude towards AIDS and patients afflicted with the disease, an outcome of 1.93 ± 0.75 out of 4 was observed. The results suggest a significant lack of knowledge about HIV, especially about the different modes of transmission and prevention strategies established by Centers for Disease Control and Prevention (CDC). Females showed a statistically significant better HIV knowledge base than the males.

The sample population was 22–25 years of age with 63.8% female participants and 36.2% males. Among them, 30.9% students were studying dentistry while the rest were in medical school. Table 1 contains demographic details of respondents.

**Table 1:** Demographic details.

Variable		N (%) N = 414
Age (years)	22	70 (16.9)
	23	180 (43.5)
	24	55 (13.3)
	25	109 (26.3)
Gender		
	Male	150 (36.2)
	Female	264 (63.8)
Degree		
	MBBS	286 (69.1)
	BDS	128 (30.9)

Male group (n = 150) was observed to have a mean score of 9.19 (SD = 4.37) on knowledge scale. By comparison, the female group (n = 264) was associated with a higher mean score of 10.49 (SD = 4.30). An independent samples t-test was performed to test if the difference was statistically significant. Data was adequately normal for t-test (skew < 2.0 and kurtosis < 9.0 in magnitude; Schmider, Zeigler, Danay, Beyer & Buhner, 2010). Levene’s F test satisfied the assumption of homogeneity of variances with F(412) = 2.623 and p = 0.106. T-test showed a statistically significant p-value of 0.003, t(412) = -2.95. Thus, female students showed a significantly higher level of knowledge about HIV. The similar analysis suggested no significant difference (using 0.005 p-value cut off) in attitude scores among the two groups. The students of MBBS and BDS did not show any significant difference in their knowledge or attitudes. Table 2 and Table 3 contain gender-wise knowledge and attitude scores of respondents.

**Table 2:** Gender-wise knowledge and attitude score.

Group Statistics						
	Gender	N	Mean	Std. Deviation	Std. Error Mean	p-value
Knowledge score out of 17	Male	150	9.19	4.369	.357	.003
	Female	264	10.49	4.299	.265	
Attitude score out of 4	Male	150	1.9067	.69848	.05703	.589
	Female	264	1.9470	.77326	.04759	

**Table 3:** Designation-wise knowledge and attitude score.

	Degree	N	Mean	Std. Deviation	Std. Error Mean	p-value
Knowledge score out of 17	MBBS	286	10.09	4.451	.263	.636
	BDS	128	9.87	4.178	.369	
Attitude score out of 4	MBBS	286	1.9441	.70861	.04190	.634
	BDS	128	1.9063	.82708	.07310	

As the students get advanced in their studies, their level of knowledge regarding HIV and AIDS got higher. To test this, we divided the population into two groups according to age. Younger group (n = 250) was found to have a mean score of 9.55 (SD = 4.784) on knowledge, that was 1.19 points lower than that observed in the relatively older population (10.74 with SD of 3.53). Independent samples t-test was performed after satisfying the normality and homogeneity of data. T-test showed t(412) = -2.73 with a significant p-value of 0.007. So, the difference in knowledge levels among both groups was not due to chance alone. Analysis of the attitude scores of both populations did not reveal a significant difference. Table 4 contains age-wise knowledge and attitude scores of respondents.

**Table 4:** Age-wise knowledge and attitude score.

	Age	N	Mean	Std. Deviation	Std. Error Mean	p-value
Knowledge score out of 17	22, 23	250	9.55	4.784	.303	.007
	24, 25	164	10.74	3.527	.275	
Attitude score out of 4	22, 23	250	1.9480	.75603	.04782	.599
	24, 25	164	1.9085	.73321	.05725	

It could either mean that our schooling system is adequate to raise the level of knowledge among the students through the years of training, or that as the students mature, they are keen to acquire knowledge about this disease. A similar low level of satisfactory attitude towards AIDS-afflicted patients raises many questions about the community and social education in medical and dental schools. A summary of responses categorized by demographics is shown in Table 5 with percentages.

**Table 5:** Item-wise response distribution.

		Age		Gender		Degree	
		22, 23	24, 25	Male	Female	MBBS	BDS
		Row N%	Row N%	Row N%	Row N%	Row N%	Row N%
HIV and AIDS are the same thing	True/Do not know	58.0%	42.0%	44.7%	55.3%	64.0%	36.0%
	False	61.7%	38.3%	31.4%	68.6%	72.0%	28.0%
HIV/AIDS is a disease of homosexuals or bisexuals ONLY	True/Do not know	22.6%	77.4%	28.3%	71.7%	71.7%	28.3%
	False	65.9%	34.1%	37.4%	62.6%	68.7%	31.3%
Heterosexual person cannot get HIV/AIDS	True/Do not know	75.5%	24.5%	55.3%	44.7%	67.0%	33.0%
	False	55.9%	44.1%	30.6%	69.4%	69.7%	30.3%
Coughing and sneezing do not spread HIV	False/Do not know	67.7%	32.3%	40.0%	60.0%	66.5%	33.5%
	True	56.0%	44.0%	34.0%	66.0%	70.7%	29.3%
A person can get HIV by sharing a glass of water with a person who has HIV	True/Do not know	76.6%	23.4%	41.9%	58.1%	67.7%	32.3%
	False	49.4%	50.6%	32.4%	67.6%	70.0%	30.0%
Showering or washing one's genitals/private parts, after sex keeps a person from getting HIV	True/Do not know	51.0%	49.0%	42.3%	57.7%	68.4%	31.6%
	False	68.8%	31.2%	30.7%	69.3%	69.7%	30.3%
A person can get HIV through contact with saliva, tears, urine or sweat	True/Do not know	65.2%	34.8%	35.2%	64.8%	70.0%	30.0%
	False	53.3%	46.7%	37.7%	62.3%	67.7%	32.3%
A person can get HIV after having oral sex	False/Do not know	65.5%	34.5%	42.7%	57.3%	66.5%	33.5%
	True	55.3%	44.7%	29.8%	70.2%	71.6%	28.4%
A person can get HIV if he/she has sex only once in his/her life	False/Do not know	75.2%	24.8%	40.1%	59.9%	68.8%	31.2%
	True	51.4%	48.6%	33.9%	66.1%	69.3%	30.7%
A person can get HIV if he/she has sex with only one partner	False/Do not know	79.1%	20.9%	37.3%	62.7%	69.5%	30.5%
	True	46.4%	53.6%	35.4%	64.6%	68.8%	31.2%
All pregnant women infected with HIV will have babies born with AIDS	True/Do not know	59.8%	40.2%	45.3%	54.7%	65.4%	34.6%
	False	61.0%	39.0%	26.5%	73.5%	73.0%	27.0%
People who have been infected with HIV quickly develop signs of severe disease	True/Do not know	62.3%	37.7%	49.7%	50.3%	67.5%	32.5%
	False	59.3%	40.7%	28.5%	71.5%	70.0%	30.0%
A person with HIV can look and feel normally	False/Do not know	50.9%	49.1%	28.9%	71.1%	70.2%	29.8%
	True	64.0%	36.0%	39.0%	61.0%	68.7%	31.3%
There is a vaccine that can stop adults from getting HIV	True/Do not know	64.1%	35.9%	38.8%	61.2%	69.6%	30.4%
	False	53.2%	46.8%	31.2%	68.8%	68.1%	31.9%
People are likely to get HIV by deep kissing if their partner has HIV	False/Do not know	67.4%	32.6%	36.0%	64.0%	69.2%	30.8%
	True	33.7%	66.3%	37.2%	62.8%	68.6%	31.4%
A person will not get HIV if he or she is taking antibiotics	True/Do not know	57.9%	42.1%	40.1%	59.9%	73.0%	27.0%
	False	61.8%	38.2%	34.0%	66.0%	66.8%	33.2%
Having sex with more than one partner can increase a person's chance of getting infected with HIV	False/Do not know	73.7%	26.3%	52.6%	47.4%	70.2%	29.8%
	True	58.3%	41.7%	33.6%	66.4%	68.9%	31.1%

Are you willing to live with people having HIV/AIDS in the same community?	Yes (45%)
Do you dislike having physical contact with HIV/AIDS patients?	312"> Yes (61.4%)
Do you accept HIV-infected students or colleagues in the same workplace or classroom?	312"> Yes (64.8%)
Caring for a patient with HIV would make me feel uncomfortable	312"> No (37.1%)

## Discussion

Adequate knowledge of healthcare professionals is crucial for the care of HIV/AIDS patients, and it also depicts the level of education and counselling that they would provide to their patients. A positive attitude towards patients with PLWHA is essential for clinicians to enhance the outcomes of treatment and prevention strategies. Physicians can play a crucial role to abridge a wide gap of knowledge, attitudes and practices (KAP) between the educated and uneducated population with AIDS [6]. Dental students are in close patient contact and assessment of their KAP towards HIV (KAP-HIV) is as important as that of medical students [7].

According to our study, it is evident that knowledge and behaviour of future healthcare professionals, i.e. medical and dental students, is poor regarding HIV/AIDS: score of 10.02 ± 4.37 out of 17 for knowledge of HIV/AIDS and 1.93 ± 0.75 out of 4 for acceptable attitude towards PLWHA. This lack of knowledge can be attributed to the inadequate educational system. A previous study done in Karachi, Pakistan also found a high knowledge deficit in medical students regarding HIV and its modes of transmission [8]. An integrated system of education can provide a better understanding of the disease. Dedicated lectures focused on teaching students the ethical aspects of dealing with HIV patients could be very helpful. All of this will lead to sound knowledge and resultant professional attitude towards AIDS-afflicted patients.

Knowledge and behaviour of medical and dental students among males (36.2%) and females (63.8%) vary. Our study showed that women have a better understanding than men: score of 10.49 ± 4.30 for women and 9.19 ± 4.37 for males, with a p-value of 0.003. The reason behind this difference can be the fact that females take their studies more seriously and show keenness in acquiring knowledge. Though in a similar analysis, no significant difference in behaviour towards PLWHA was found between these two populations.

The study also revealed that as the students grow older, their knowledge of disease increases: 10.74 ± 3.53 for the older group (aged 24–25 years old) and 9.55 ± 4.784 for the younger group (aged 22–23 years old), with a p-value of 0.007. This can be explained by the fact that the older group has gained more experience in the field over the years and has been exposed to a larger number of patients as compared to the younger group. Also, after working in the clinical setting in the senior years of medical school, the older group have a better understanding of the disease process as compared to the younger group with no or minimal clinical exposure. However, analysis of attitude scores for these two groups did not show any difference in behaviour towards HIV/AIDS patients. This negative attitude is consistent with taboos and social practices in our society. HIV-related stigma is real [9,10]. It increases the fear of acquiring the infection, decreases the level of empathy towards HIV patients on mass levels and grows a discriminatory attitude that is not appropriate for a healthcare professional [11,12]. Irritability, anxiety, depression and psychological reactance have been associated with HIV-related stigma, especially in women. Healthcare professionals need to curtail their deficiencies in knowledge, attitudes and practices regarding HIV/AIDS to address these problems [10].

High-quality educational programs have been tested in the western world to raise the level of positive attitudes and knowledge regarding HIV in healthcare professionals [13,14]. Therefore, regular interactive workshops and seminars, besides teaching sessions, focused lectures on HIV/AIDS, need to be conducted. Betterment of educational system and curriculum with standardisation are warranted to improve this alarming situation. Moreover, there is a need for regular and massive public education campaign to enhance awareness about the disease.

## Conclusions

Keeping in mind the worldwide prevalence, morbidity, and mortality of HIV/AIDS and the fact that the current medical and dental students will ultimately take care of these patients, it is clear from this study that both of these groups are not equipped with some fundamental information and have negative attitudes towards the disease. It is essential that all aspects of the disease, in general, and some, in particular, be targeted to make sure that every student is well-versed with at least the necessary information. Regular workshops and seminars on HIV/AIDS awareness need to be organised to better educate students about this disease.
